# Dataset of mountain pine beetle outbreak dynamics and direct control in Cypress Hills, SK

**DOI:** 10.1016/j.dib.2020.105293

**Published:** 2020-02-17

**Authors:** Mélodie Kunegel-Lion, Rory L. McIntosh, Mark A. Lewis

**Affiliations:** a Department of Biological Sciences, University of Alberta, CW 405 Biological Sciences Bldg, Edmonton, AB, T6G 2E9, Canada; b Forest Service Branch, Saskatchewan Ministry of Environment, Box 3003 McIntosh Mall, Prince Albert, SK, S6V 6G1, Canada; c Mathematical and Statistical Sciences, University of Alberta, 632 CAB, Edmonton, AB, T6G 2G1, Canada

**Keywords:** Insect epidemics, Pest management, Direct control, Simulations

## Abstract

The data presented in this article are related to the research article entitled “Mountain pine beetle outbreak duration and pine mortality depend on direct control effort” [1]. This article provides presence of mountain pine beetle infested trees detected by the Saskatchewan Forest Service on a grid covering the spatial extent of the Saskatchewan portion of the Cypress Hills interprovincial park between 2006 and 2018. For each grid cell, associated ecological and environmental covariates, such as topography, weather and vegetation, are also provided. These data cover the spatio-temporal extent of an almost entire mountain pine beetle outbreak and contribute to the understanding of mountain pine beetle outbreak dynamics.

**Table:** Specifications Table

Subject	Ecology
Specific subject area	Insect outbreak dynamics
Type of data	Tables
How data were acquired	Aerial and ground surveys, collection of data across literature and databases
Data format	Raw
Parameters for data collection	Data were collected between 2006 and 2018 in the Saskatchewan portion of Cypress Hills interprovincial park, Canada, which correspond to the spatial and temporal extent of the focus mountain pine beetle outbreak.
Description of data collection	Mountain pine beetle infested trees were identified and located by the Saskatchewan Environment Forest Service. We estimated weather variables for each year using the software BioSIM. We obtained topography data through GeoGratis.gc.ca. We interpolated vegetation data from Ref. [2].
Data source location	Cypress Hills Interprovincial Park, Saskatchewan, Canada
Data accessibility	Repository name: Dryad Data identification number: 10.5061/dryad.70rxwdbt9 Direct URL to data: https://doi.org/10.5061/dryad.70rxwdbt9
Related research article	Mélodie Kunegel-Lion & Mark A. Lewis, Mountain pine beetle outbreak duration and pine mortality depend on direct control effort, Journal of Environmental Management, DOI: 10.1016/j.jenvman.2020.110167

**Table:** 

**Value of the Data** • These data are useful for understanding the fine-scale dynamics of an almost complete mountain pine beetle outbreak limited in its spatial range. • Researchers and managers can benefit from these data to understand outbreak dynamics or to inform decision-making processes and management strategies. • These data can be used to further insight spatial dynamics of dispersal and can be used for modelling.

## 1 Data description

The data presented in this article are related to the research article entitled “Mountain pine beetle outbreak duration and pine mortality depend on direct control effort” [1]. Data are provided for each year between 2006 and 2018 and for each cell of a grid covering the spatial extent of the study area. Two datasets are provided for two grid spatial resolution: a grid of 18,317 100 × 100 m cells (dataset 1) and a grid of 722 500 × 500 m cells (dataset 2). For each cell of a grid, the dataset contains the presence or number of mountain pine beetle infested trees as well as topography, weather, vegetation, and management factors (Table 1). The topography factors included are latitude, longitude, elevation, slope, aspect, northerness, easterness. The weather factors included are maximum temperature in summer, minimum temperature in summer, minimum temperature over the year, degree-days, cold tolerance, relative humidity in spring, soil moisture index, wind speed in summer, and an estimate of mountain pine beetle emergence peak from degree-days. The vegetation factors include pine cover, pine height, pine age, and number of pines. The management factors included are presence of managed and unmanaged mountain pine beetle infested trees the previous year in the same cell and in neighbouring cells, and the distance between the cell and a limited patch of unmanaged infestations outside the park's southern limit.

**Table 1:** Description of the variables.

Variable	Description	Unit
MPB	Presence (dataset 1) or number (dataset 2) of pine trees currently infested by living mountain pine beetle eggs or larvae	pines
Latitude	Latitude of the grid cell centroid	dec.°
Longitude	Longitude of the grid cell centroid	dec.°
Elevation	Elevation at the grid cell centroid	m
Slope	Slope at the grid cell centroid	°
Aspect	Compass direction the slope faces	°
Northerness	Spatial property of a slope to face North	–
Easterness	Spatial property of a slope to face East	–
TMax	Highest maximum daily temperature during July and August	°C
TMin_Summer	Lowest minimum daily temperature during July and August	°C
TMin_Winter	Lowest minimum daily temperature in the winter	°C
DegreeDays	Degree-days above 5.5°C from the fall of the previous year to the summer	–
ColdTolerance	Cold tolerance estimates the probability of larva survival over the winter using temperature	%
RelativeHumidity	Average relative humidity from March to May	%
SMI	The average soil moisture index from May to July estimates soil water availability for tree growth using temperature and precipitation (only available for dataset 1)	mm
WindSpeed	Average wind speed in July and August	km/h
PeakEmergence	Mountain pine beetle emergence peak estimates when 50% of the beetles have emerged from cumulative degree-days above 2°C starting on May 30th (Julian day 150; only available for dataset 1)	Julian day
PineCover	Coverage of *Pinus albicaulis* (whitebark pine), *Pinus banksiana* (jack pine) and *Pinus contorta* (includes subspecies lodgepole pine and shore pine)	%
PineHeight	Height of the dominant tree species in the cell when the pine cover is greater than 50%	m
PineAge	Age of the dominant tree species in the cell when the pine cover is greater than 50%	year
Stems	Number of pine trees greater than 10 cm at breast height	pines
BP0	Presence (dataset 1) or number (dataset2) of previous-year mountain pine beetle infested trees in the cell	–
BP0man	Presence of previous-year controlled mountain pine beetle infested trees in the cell and no uncontrolled trees (dataset 1) or number of previous-year controlled infested trees in the cell (dataset 2)	–
BP0red	Presence (dataset 1) or number (dataset 2) of previous-year uncontrolled mountain pine beetle infested trees in the cell	–
BP1	Number of infested cells (dataset 1) or number of infested trees (dataset 2) the previous year in a 1-cell radius excluding the focus cell	
BP1man	Number of infested cells with all trees controlled (dataset 1) or number of infested trees (dataset 2) the previous year in a 1-cell radius excluding the focus cell	–
BP1red	Number of infested cells with uncontrolled trees (dataset1) or number of uncontrolled infested trees (dataset 2) the previous year in a 1-cell radius excluding the focus cell	–
BP2	Number of infested cells (dataset 1) or number of infested trees (dataset 2) the previous year between a 1-cell and 2-cell radius	–
BP2man	Number of infested cells with all trees controlled (dataset 1) or number of infested trees (dataset 2) the previous year between a 1-cell and 2-cell radius	–
BP2red	Number of infested cells with uncontrolled trees (dataset1) or number of uncontrolled infested trees (dataset 2) the previous year between a 1-cell and 2-cell radius	–
BP3	Number of infested cells (dataset 1) or number of infested trees (dataset 2) the previous year between a 2-cell and 3-cell radius	–
BP3man	Number of infested cells with all trees controlled (dataset 1) or number of infested trees (dataset 2) the previous year between a 2-cell and 3-cell radius	–
BP3red	Number of infested cells with uncontrolled trees (dataset1) or number of uncontrolled infested trees (dataset 2) the previous year between a 2-cell and 3-cell radius	–
Dist2borderS	Distance from the centre of the cell to the park’s southern limit close to external infestations	m

## 2 Experimental design, materials, and methods

### 2.1 Mountain pine beetle infested trees

Saskatchewan Ministry of Environment Forest Service is responsible for surveying mountain pine beetles in this portion of the park. Every year, a complete aerial survey of the park extent is performed in order to detect red-top trees [3]. These are later ground-truthed for beetle attacks and trees fading from other causes than mountain pine beetle are removed from the survey. Ground surveys with a radius of 50 m are conducted around each red-top tree containing beetles to find currently infested pines with live brood. However, in some areas, red-top trees are clustered together. To thoroughly survey these areas, polygons are delineated by hand around each red-top trees cluster. The extent of each polygon is then entirely checked for infested trees using line surveys. Polygon locations and shapes typically change from one year to the other. However, they are consistently located for the most part in the same highly infested and therefore highly surveyed areas. Infested pines located outside of the circular plots or polygons would be most likely missed by managers. All detected infested trees are treated, primarily using a fall and burn tactic to ensure that the broods are killed. The Forest Service has been following this procedure since the mountain pine beetle infestation was detected in 2006 up to the current state of the outbreak in 2018. In summary, we obtained the following data for each year: locations of red-top trees, locations and shapes of polygons, number of infested trees for each circular survey, and locations of infested trees within the polygons. From this information, we estimated the locations of all infested trees.

To get an estimate of all of the infested trees location for every year, we used the following methods.

• For the controlled infested trees found in circular survey plot (called type C1), the exact coordinates of the infested trees were not recorded so we used the location of the plot's centroid.
• For the controlled infested trees found in line surveys (*i.e.* within polygons, called type C2), we used the exact location of the infested trees.
• For the uncontrolled infested trees that were missed at year t, became red-top trees at year t+1 and were not part of a polygon at year t+1 (called type U1), we used the exact location of the red-top trees at year t+1.
• For the uncontrolled infested trees that were missed at year t, became red-top trees at year t+1 and were part of a polygon at year t+1 (called type U2), the exact coordinates of red-top trees were not recorded. We first assumed that, in Cypress Hills, the proportion of uncontrolled infested trees within these highly surveyed areas is 0.11 [4]. Therefore, the proportion of controlled infested trees within ground survey areas is 1–0.11 = 0.89. Note that this number is different from the proportion of controlled infested trees in the entire park as ground surveys do not cover the entire park extent. Using these proportions, the number of controlled trees in polygons is equal to 0.89 times the number of infested trees. So, the number of infested trees in polygons is the number of controlled trees in polygons divided by 0.89. Therefore, the number of uncontrolled infested trees in polygons is

# uncontrolled infested trees = 0.11 × # infested trees = 0.11 × # controlled infested trees /0.89

Second, using the equation above, we modelled, for each t+1 polygon, the number of infested trees that would have been missed and fell within the polygon areas at year t+1 as a Poisson random variable with mean equal to the number of uncontrolled infested trees at t that fall into a t+1 polygon.

Third, we randomly distribute in space the U2 infested trees in each polygon area.

**Fig. 1:**
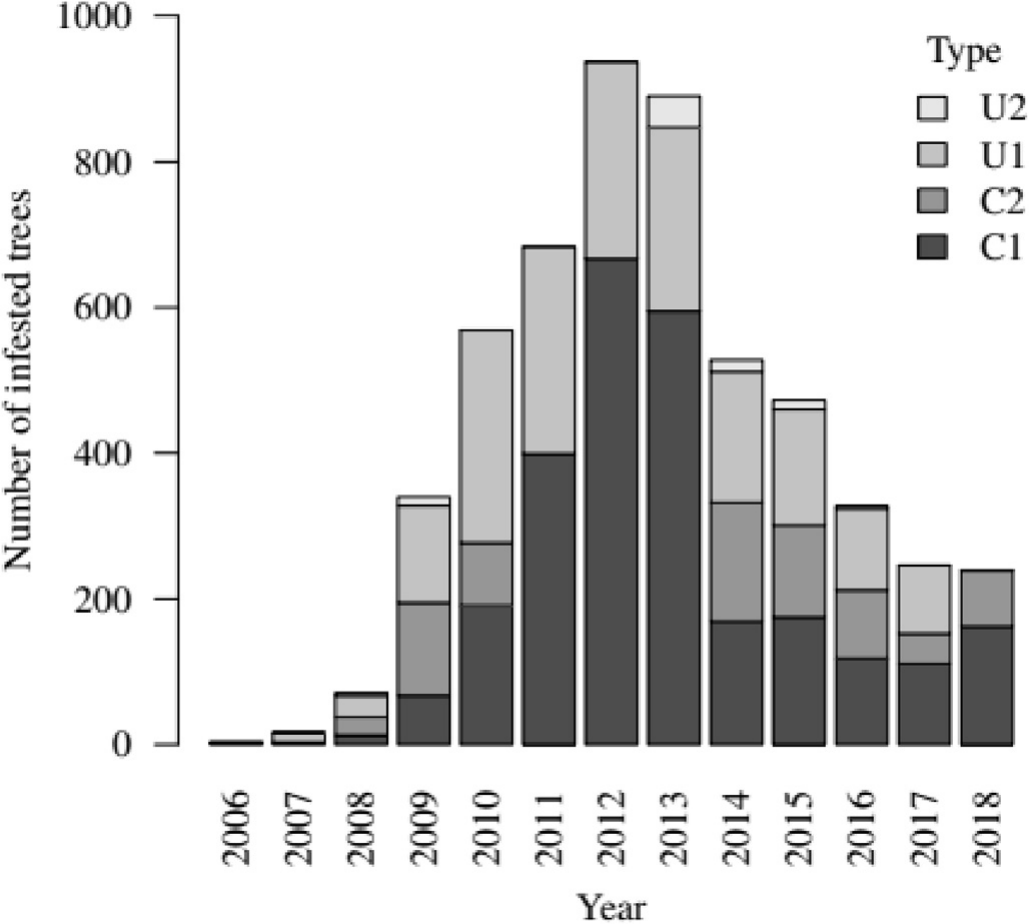
Number of infested trees per type over the years in Cypress Hills, SK. Type C1 represents the controlled infested trees found in circular surveys whereas type C2 represents the controlled infested trees found in line surveys. Types U1 and U2 represent the uncontrolled infested trees at year t that become red-top trees, respectively, outside or inside of a year-t+1 polygon. For the year 2018, the numbers of U1 and U2 infested trees are unknown at the time of the study as they can only be estimated with 2019 data.

In total, from 2006 to 2018, we had 2672 trees of type C1, 740 trees of type C2, 1819 trees of type U1, and 93 trees of type U2 (Fig. 1). These numbers are consistent with the previously estimated control efficiency over the entire park extent: 60.6% and 73.7% of infested trees were controlled in 2011 and 2012, respectively [4]. We were able to estimate the location of all infested trees every year with a precision of a few meters for 48% of the infested trees (types C2 and U1), 50 m for 50% of the infested trees (type C1), and several hundred meters depending on the polygons size for the remaining 2% (type U2). Finally, a grid with cell size 100 × 100 m or 500 × 500 m is superimposed over the park extent and the presence or number of infested trees in each cell is recorded.

### 2.2 Ecological and environmental covariates

We obtained topography variables from the Canadian Digital Elevation Map downloaded from the Geogratis website (geogratis.cgdi.gc.ca). We extracted the elevation, slope, and aspect. The aspect was converted into northerness and easterness using, respectively, a cosine and sine transformation. We estimated weather variables at the centroid of each grid cell for each year using the software BioSIM and the associated weather station data [5]. We obtained an estimate of the mountain pine beetle emergence peak each year by using the model and parameters provided in Ref. [6] (model 1) and cumulative degree-days from BioSIM [5].

From Ref. [2], we estimated, for each cell in 2001 and 2011, the leading species height, the pine cover and the tree volume. Then, we obtained pine height by using the leading species height when the pine cover is greater than 50%. We spatially interpolated the pine height values at the location of each cell using bicubic spline interpolation provided by the function interp of the R package akima [7]. We obtained the pine volume by multiplying the pine cover by the tree volume. To obtain vegetation variable values for every year, we linearly interpolated the values over the time period for each cell. We estimated the number of pine trees in each cell from the pine volume using the process and equation described in Ref. [8]. The expected number of pines greater than 10 cm at breast height E(S) depends on the pine volume per hectare V following equation E(S) = A V exp(-δ V) where A and δ are free parameters. Pines with a diameter at breast height smaller than 10 cm are seldom the target of mountain pine beetle attacks and therefore were not included in the pine count [9]. Based on [10], Cypress Hills has a site index of 15–18 m at 50 year breast height age for lodgepole pine which corresponds to a medium to good site in Ref. [11]. Therefore, we fit A and δ to data from the yield tables for medium and good sites provided in Ref. [11] using a nonlinear regression and obtained the values A = 18.18 (±0.23 SE) and δ = 5.4 × 10^−3^ (±0.4 × 10^−3^ SE).
